# Exogenous Plant Growth-Promoting Rhizobacteria Enhance the Promoting Effect of Polyaspartic Acid on Potato Growth by Improving Rhizosphere Nutrient Availability and Reshaping Microbial Community

**DOI:** 10.3390/plants14223530

**Published:** 2025-11-19

**Authors:** Xin Zhou, Xia Zhu, Xiangquan Fan, Xueli Huang, Haiyan Ma, Hafsa Nazir Cheema, Kaiqin Zhang, Shunlin Zheng

**Affiliations:** 1 State Key Laboratory of Crop Gene Exploration and Utilization in Southwest China, College of Agronomy, Sichuan Agricultural University, Chengdu 611130, China; 18583961594@163.com (X.Z.); hxueli1983@163.com (X.H.); hafsanazeer55@hotmail.com (H.N.C.); zhangkaiqin123456@163.com (K.Z.); 2 Crop Ecophysiology and Cultivation Key Laboratory of Sichuan Province, Chengdu 611130, China; 3 Shannan Municipal Agricultural Technology Extension Centre, Shannan 856000, China; zhuxia8525@163.com; 4 Dazhou Academy of Agricultural Sciences, Dazhou 635000, China; dzfxquan@163.com; 5 Yibin Academy of Agricultural Sciences, Yibin 644699, China; mahaiyanjinji@gmail.com; 6 Key Laboratory of Tuber Crop Genetics and Breeding, Ministry of Agriculture, Chengdu Joyson Agricultural Technology Co., Ltd., Chengdu 610500, China

**Keywords:** potato, plant growth-promoting rhizobacteria, Polyaspartic acid, soil nutrients, rhizosphere microbiome, sustainable agriculture

## Abstract

Polyaspartic acid (PASP), a biodegradable and eco-friendly fertilizer synergist that shows potential to enhance nutrient use efficiency in agricultural systems, has its integrative role with rhizosphere microorganisms remain insufficiently explored. This study integrated outdoor pot experiments, soil biochemical analysis, and microbiome sequencing to investigate the effects of co-application of PASP and the plant growth-promoting rhizobacterium (PGPR) *Enterobacter asburiae* S13 on potato growth, with four treatments set up including blank control (CK), sole application of PASP (S0P1), sole inoculation of PGPR (S1P0), and co-application of PASP and PGPR (S1P1), and 25 pots per treatment as replicates. The results showed that, compared with the S0P1 treatment, the S1P1 treatment significantly increased plant height (9.59%), stem diameter (28.39%), root length (38.61%), as well as root and shoot biomass (21.26% and 25.17%, respectively) (ANOVA, Duncan’s test, *p* < 0.05). It also enhanced ammonium nitrogen (40.00%), nitrate nitrogen (57.70%), available potassium (47.56%), and urease activity in the rhizosphere soil (ANOVA, Duncan’s test, *p* < 0.05). 16S rRNA sequencing revealed that the S1P1 treatment enriched beneficial taxa such as *Paucibacter* and *Massilia*, while suppressing competitive genera such as *Duganella* and *Pedobacter*. Redundancy analysis (RDA) indicated that available potassium and ammonium nitrogen were the key factors shaping the microbial community structure. In conclusion, combining PASP with PGPR synergistically improves soil nutrient availability and reshapes the rhizosphere microbiome, resulting in enhanced potato growth, thus demonstrating its potential as a dual-function biostimulant for eco-efficient and sustainable potato production systems.

## 1. Introduction

Potato (*Solanum tuberosum* L.), the fourth-largest staple crop globally, with a cultivation area of approximately 18.9 million hectares [1], plays a crucial role in ensuring food security and sustainable agricultural development. In China, efforts to enhance potato yield have historically relied on intensive fertilizer and pesticide applications, which exacerbate environmental issues such as soil degradation, declining fertility, and non-point source pollution [2]. Addressing these challenges requires eco-friendly strategies to optimize nutrient use and improve soil health.

Polyaspartic acid (PASP), a biodegradable amino acid-based polymer naturally found in snails and mollusks, can also be synthesized through the thermal polycondensation of aspartic acid [3]. As a fertilizer synergist, PASP has shown considerable potential in enhancing crop growth and yield by chelating rhizosphere nutrients [4,5], stimulating microbial activity [6], and regulating plant metabolism via its degradation product, aspartic acid [4]; notably, it has been reported to improve nitrogen assimilation pathways in potatoes, promoting growth under low-nitrogen conditions [7]. Additionally, the molecular weight and degradation kinetics of PASP may influence rhizosphere nutrient release, microbial activity, and its compatibility with microbial inoculants [4,6]. However, despite existing studies, the synergistic effects of PASP with microbial inoculants, particularly plant growth-promoting rhizobacteria (PGPR), remain unexplored.

PGPR are root-colonizing microorganisms that promote plant growth through various mechanisms [8], including nutrient solubilization and modulation of soil–plant-microbe interactions [9,10]. For example, phosphate- and silicate-solubilizing strains enhance the availability of essential elements in the rhizosphere [11], while nitrogen-fixing bacteria improve plant nitrogen assimilation efficiency [12]; notably, *Enterobacter* species, as typical PGPR, exhibit distinct specific functions, such as producing siderophores to chelate environmental iron and enhance its bioavailability, and secreting plant hormones like indole-3-acetic acid (IAA) to directly regulate plant growth and development [13]. PGPR that colonize mineral surfaces proliferate by forming biofilms composed of extracellular polymers, such as cellulose, proteins, and galactose, while releasing metabolites, including organic acids, proteins, and enzymes [14,15]. The dynamic chemical reactions within these biofilms create favorable conditions for PGPR to assimilate inorganic nutrients, facilitating mineral weathering and enhancing plant nutrient absorption [14]. The restructuring of the rhizosphere microbial community is primarily driven by changes in the composition and concentration of soil exudates. Exogenously applied PGPR influence interspecies interactions through their secreted products and can also induce host plants to alter the composition and quantity of root exudates [16,17]. This modulation recruits complementary microbial taxa, collectively promoting plant growth.

The combined use of growth-promoting agents has emerged as an effective strategy to enhance crop resilience and productivity [18]. For instance, the combination of biochar and nano-silicon improves drought resistance in wheat (*Triticum aestivum* L.) [19], while microbial fertilizers integrated with biochar enhance soil phosphorus and potassium fertility, promoting tomato (*Solanum lycopersicum* L.) growth [8]. Despite studies highlighting PASP’s regulatory effects on the rhizosphere microbiome—such as reshaping microbial communities and increasing rhizosphere microbial diversity [20], the effects of PGPR inoculation on rhizosphere nutrient dynamics, microbial community structure, and plant performance—particularly under PASP-supplemented cultivation systems—remain underexplored.

A pot experiment was conducted to assess the effects of PGPR (*Enterobacter asburiae* S13) inoculation combined with PASP application on potato growth, rhizosphere nutrient content, and microbial community composition. We hypothesized that PGPR inoculation would synergize with PASP to (1) increase potato biomass, (2) enhance rhizosphere nutrient availability, and (3) shift the microbial community toward beneficial taxa. This study seeks to clarify the interactions among plants, soil, and microorganisms following the combined application of PASP and PGPR, offering a foundation and practical insights for developing sustainable agricultural strategies that integrate fertilizer synergists with microbial inoculants.

## 2. Results

### 2.1. Effects of PGPR Inoculation on Potato Growth

**Figure 1:**
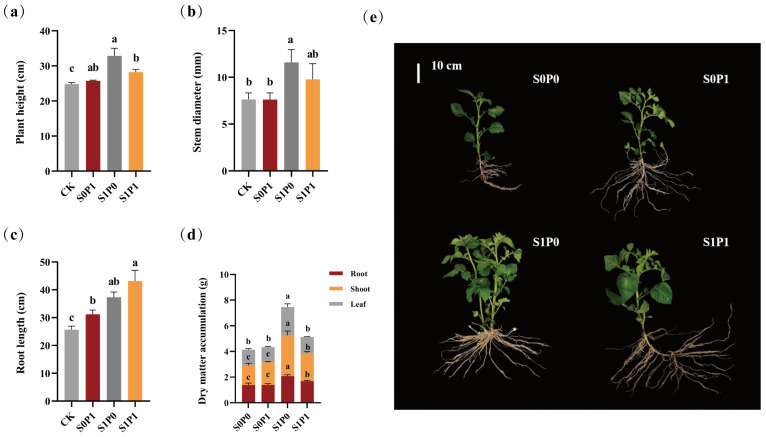
Effects of PGPR inoculation on potato growth under PASP application: (**a**) plant height (cm), (**b**) stem diameter (mm), (**c**) root length (cm), (**d**) dry weight partitioning (roots, stems, leaves) (g plant^−1^) and (**e**) phenotype of potatoes. Data are presented as means ± SD (*n* = 6). Lowercase letters indicate significant differences among treatments (ANOVA, Duncan’s test, *p* < 0.05). CK: Control (no treatment); S0P1: PASP only; S1P0: PGPR only; S1P1: PASP + PGPR.

To assess the effects of PGPR inoculation on the growth of potatoes under PASP application, we measured growth indices and the dry weights of various plant parts. Specifically, compared with the S0P1 treatment, the S1P1 treatment showed a 9.59% increase in plant height (S0P1: 25.73 ± 0.21 cm, S1P1: 28.20 ± 0.82 cm; *p* < 0.05) (Figure 1a); a 28.39% increase in stem diameter (S0P1: 7.62 ± 0.71 mm, S1P1: 9.78 ± 1.68 mm; *p* < 0.05) (Figure 1b); and a 38.61% increase in root length (S0P1: 31.17 ± 1.55 cm, S1P1: 43.20 ± 3.81 cm; *p* < 0.05) (Figure 1c). The S1P1 treatment also notably enhanced the biomass of both roots and stems (*p* < 0.05): relative to the S0P1 treatment (root dry weight: 1.38 ± 0.12 g; stem dry weight: 1.76 ± 0.07 g), the dry weights of roots and stems in S1P1 increased by 21.26% (S1P1: 1.68 ± 0.07 g) and 25.17% (S1P1: 2.20 ± 0.11 g), respectively (Figure 1d).

### 2.2. Effects of PGPR Inoculation on Rhizosphere Soil Biochemical Properties

**Figure 2:**
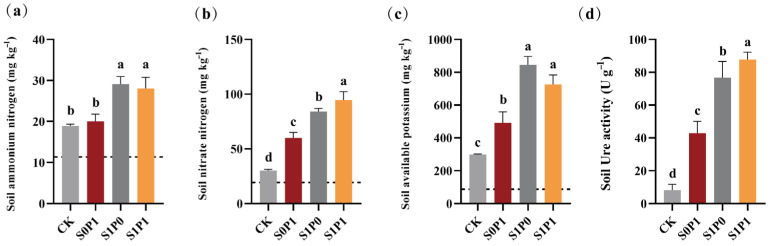
Effect of PGPR inoculation on soil biochemical properties under PASP application: (**a**) ammonium nitrogen (NH_4_^+^-N) (mg kg^−1^), (**b**) nitrate nitrogen (NO_3_^−^-N) (mg kg^−1^), (**c**) available potassium (AK) (mg kg^−1^), and (**d**) soil urease (S-Ure) activity (U g^−1^). Dashed lines in the figure indicate the baseline values of the respective indicators. Data are presented as means ± SD (*n* = 6). Different letters indicate significant differences (ANOVA, Duncan’s test, *p* < 0.05). CK: Control (no treatment); S0P1: PASP only; S1P0: PGPR only; S1P1: PASP + PGPR.

To assess the effects of PGPR inoculation under PASP application on rhizosphere soil chemistry and enzyme activity, we measured soil nutrient content and urease activity. Compared to the S0P1 treatment, the S1P1 treatment significantly enhanced ammonium nitrogen (NH_4_^+^-N) (Figure 2a), nitrate nitrogen (NO_3_^−^-N) (Figure 2b), available potassium (AK) (Figure 2c), and soil urease (S-Ure) activity (Figure 2d) in the rhizosphere, increased NH_4_^+^-N, NO_3_^−^-N, and AK by 40.00%, 57.70%, and 47.56%, respectively, with all differences being statistically significant (*p* < 0.05).

### 2.3. Effects of PGPR Inoculation on Rhizosphere Microbial Community Structure and Function

**Figure 3:**
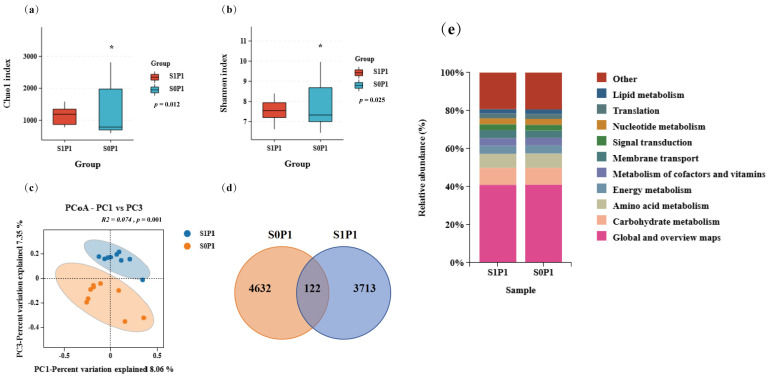
Effect of PGPR inoculation on the rhizosphere microbial community structure and function under PASP application: (**a**) α-diversity (Chao1 index, *p* = 0.012, * *p* < 0.05), (**b**) α-diversity (Shannon index, *p* = 0.025, * *p* < 0.05), (**c**) Principal Coordinate Analysis (PCoA) based on Bray–Curtis dissimilarity, (**d**) Venn diagram of OTU distribution, and (**e**) KEGG pathway composition of the top 10 functional categories (Level 2). S0P1: PASP only; S1P1: PASP + PGPR.

To further assess the effect of PGPR on the rhizosphere soil microenvironment under PASP application, we conducted 16S rRNA sequencing for the S0P1 and S1P1 treatments. Results showed that PGPR inoculation under PASP application altered the bacterial community diversity. Specifically, PGPR inoculation under PASP application decreased α-diversity indices: the Shannon index showed a significant reduction (*p* = 0.025) and the Chao1 index also decreased significantly (*p* = 0.012) (both *p* < 0.05) (Figure 3a,b). Principal Coordinate Analysis (PCoA) based on Bray–Curtis dissimilarity indicated a significant and biologically suggesting a reorganization of the microbial community structure (*p* = 0.001) (Figure 3c). Venn analysis of operational taxonomic units (OTU) abundance showed that the PGPR-inoculated group under PASP application had fewer unique OTUs (3713) compared to the PASP-alone group, with only 122 shared OTUs (Figure 3d). However, KEGG pathway analysis did not show significant changes in microbial metabolic functions (Figure 3e).

### 2.4. Effects of PGPR Inoculation on Rhizosphere Microbial Composition

**Figure 4:**
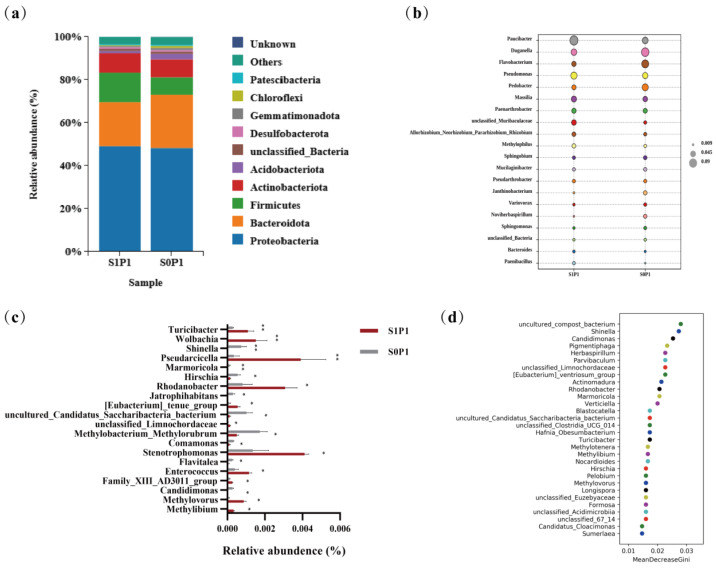
Taxonomic shifts in rhizosphere microbial composition induced by PGPR inoculation under PASP application: (**a**) phylum-level relative abundance (top 10 taxa), (**b**) genus-level composition (top 20 taxa; bubble size reflects abundance), (**c**) differentially abundant genera (top 20 taxa; asterisks indicate significance: * *p* < 0.05, ** *p* < 0.01), and (**d**) random forest analysis of the top 30 genera. S0P1: PASP only; S1P1: PASP + PGPR.

**Figure A1:**
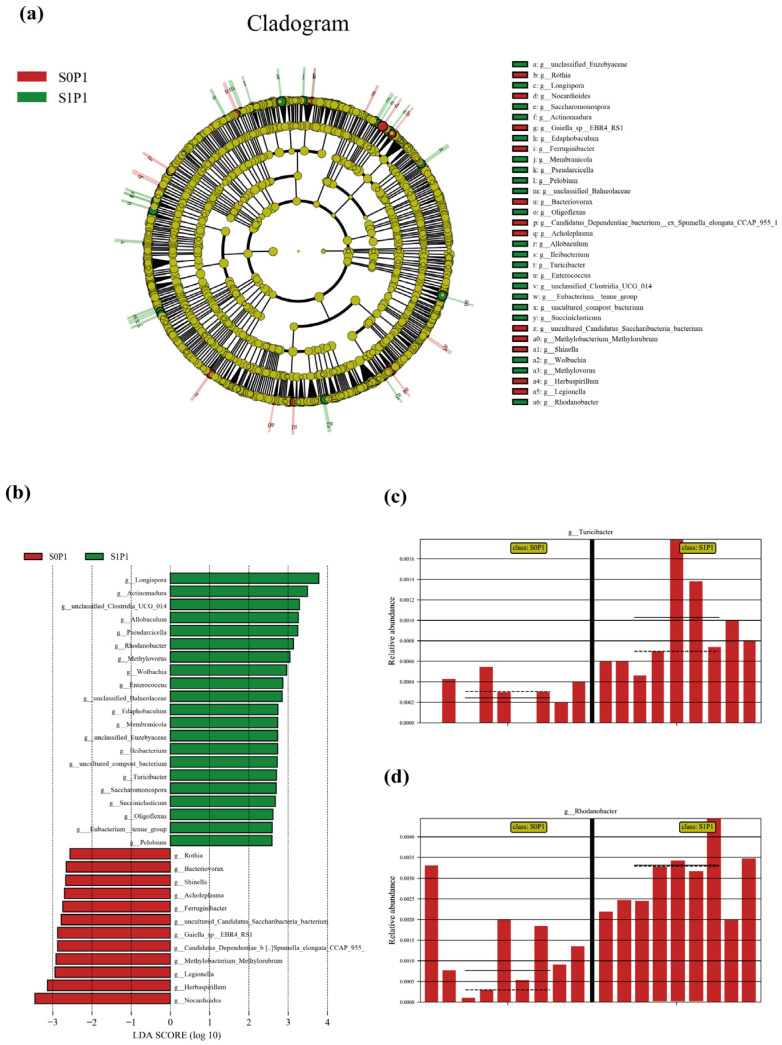
Linear Discriminant Analysis Effect Size (LEfSe) analysis to verify the robustness of taxonomic shifts in rhizosphere microbial communities (LDA score threshold = 3.5): (**a**) cladogram illustrating differentially abundant taxa between S0P1 and S1P1 groups, (**b**) LDA score plot showing the effect size of discriminative taxa, (**c**) relative abundance of *Turicibacter*, and (**d**) relative abundance of *Rhodanobacter*. S0P1: PASP only; S1P1: PASP + PGPR.

At the phylum level, PGPR inoculation increased the relative abundances of *Bacteroidota* and *Acidobacteriota* while decreasing those of *Proteobacteria*, *Firmicutes*, and *Actinobacteria* (Figure 4a). At the genus level, PGPR inoculation enriched beneficial taxa such as *Paucibacter*, *Pseudomonas*, and *Massilia*, while reducing the abundance of *Duganella*, *Flavobacterium*, and *Pedobacter* (Figure 4b). Genus-level enrichment analysis revealed distinct microbial signatures, with several taxa showing statistically significant variations in relative abundance (*p* < 0.01). Specifically, the inoculated group exhibited marked increases in *Turicibacter* and *Wolbachia*, along with significant reductions in *Shinella*, *Pseudarcicella*, and *Marmoricola*. Furthermore, taxa such as *Rhodanobacter*, *Hirschia*, and *Jatrophihabitans* displayed significant abundance differences (*p* < 0.05) (Figure 4c). Notably, genera such as *Turicibacter* (a genus associated with host metabolic regulation) and *Rhodanobacter* (a denitrification-related taxon), whose abundances increased, along with genera like *Shinella* (a potential nitrogen-cycling participant) and *Marmoricola* (a stress-responsive genus), whose abundances decreased, were identified by random forest analysis as major drivers of microbial community restructuring between treatments (Figure 4d). Linear Discriminant Analysis Effect Size (LEfSe) analysis verified the robustness of taxonomic shifts in key rhizosphere microbial genera (LDA score threshold = 3.5) (Figure A1). Meanwhile, relative abundance plots of Turicibacter and Rhodanobacter showed they were significantly discriminative between S0P1 and S1P1 treatments, with their abundance changes exhibiting relative consistency across biological replicates (Figure A1c,d).

### 2.5. Correlations Between Potato Biomass and Rhizosphere Soil Properties

**Figure 5:**
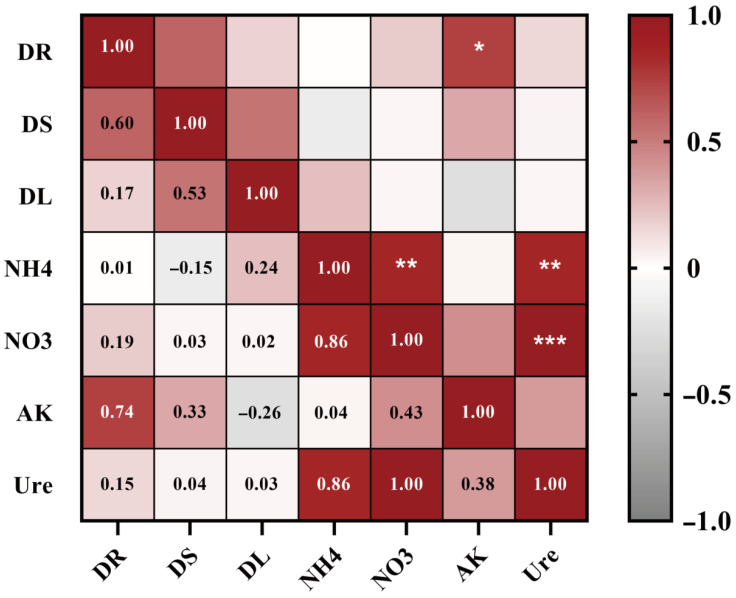
Correlation analysis between potato biomass and rhizosphere soil biochemical properties. Red and gray gradients denote positive (r > 0) and negative (r < 0) correlations, respectively. Asterisks indicate significance (* *p* < 0.05, ** *p* < 0.01, *** *p* < 0.001; Pearson’s correlation). DR: Root dry weight, DS: Stem dry weight, DL: Leaf dry weight, NH_4_: Ammonium nitrogen, NO_3_: Nitrate nitrogen, AK: Available potassium, Ure: Soil urease activity.

Correlation analysis showed that root dry weight was significantly positively correlated with AK (*p* < 0.05), whereas S-Ure activity exhibited strong positive correlations with NH_4_^+^-N (*p* < 0.01) and NO_3_^−^-N (*p* < 0.001) (Figure 5).

### 2.6. Interactions Between Soil Biochemical Properties and Microbial Diversity

**Figure 6:**
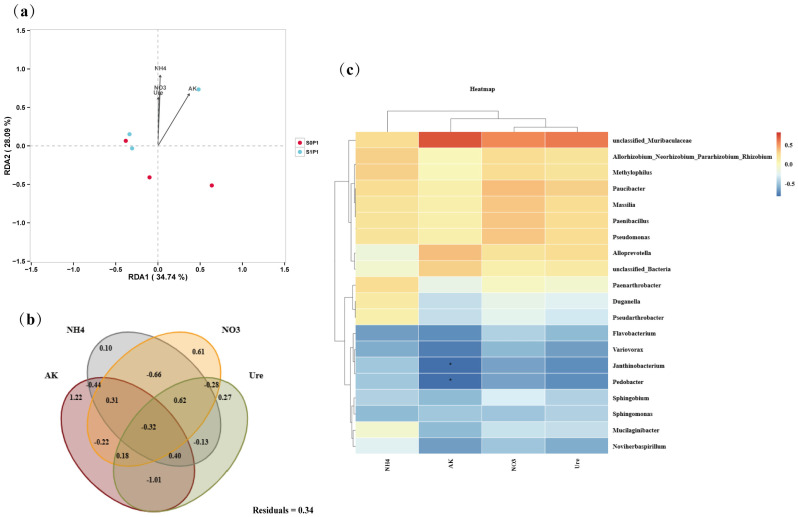
Interactions between environmental factors and microbial diversity: (**a**) redundancy analysis (RDA) of microbial communities (OTU abundance) and soil properties, (**b**) variance partitioning analysis (VPA) quantifying environmental contributions to microbial variation (Residuals: unexplained variance), and (**c**) heatmap of genus-environment correlations (top 20 genera; asterisks: * *p* < 0.05). S0P1: PASP only; S1P1: PASP + PGPR.

Redundancy analysis (RDA) of rhizosphere microbial communities and environmental factors indicated that NH_4_^+^-N content had the greatest influence on microbial community structure, followed by AK content, while NO_3_^−^-N content and S-Ure activity made marginal contributions. Notably, bacterial communities in PGPR-inoculated treatments under PASP application exhibited positive correlations with all environmental factors, whereas non-inoculated groups showed negative correlations (Figure 6a). Variance partitioning analysis (VPA) further quantified the contributions of individual environmental factors to microbial community variation. AK and NO_3_^−^-N explained 1.22% and 0.61% of the total variation, respectively. Additionally, the combined effects of NH_4_^+^-N, NO_3_^−^-N, and S-Ure activity accounted for 0.62% of the microbial community variation (Figure 6b). At the genus level, correlation analysis between dominant taxa and environmental factors revealed that beneficial genera such as *Paucibacter*, *Massilia*, and *Methylophilus* were positively correlated with nutrient availability (e.g., NH_4_^+^-N, NO_3_^−^-N, AK), whereas genera including *Duganella*, *Flavobacterium*, and *Pedobacter* showed negative correlations. Notably, *Pedobacter* and *Janthinobacterium* were negatively correlated with AK (*p* < 0.05), suggesting that these genera may be inhibited under high-potassium conditions in PASP-treated soils (Figure 6c).

### 2.7. Correlations Between Rhizosphere Soil Biochemical Characteristics and Colonization Activity of PGPR

**Figure 7:**
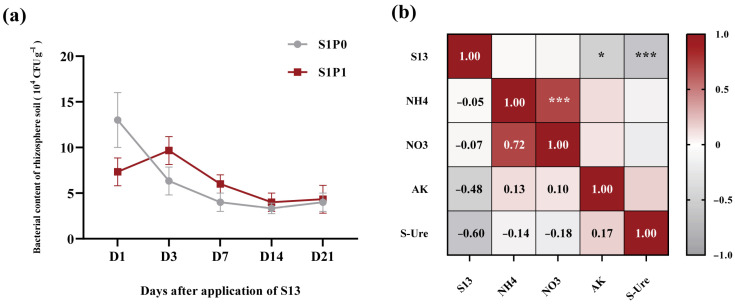
Colonization of PGPR in potato rhizospheric soil and its correlation analysis with soil biochemical properties: (**a**) PGPR content in potato rhizospheric soil (10^4^ CFU g^−1^), (**b**) correlation between PGPR content and soil biochemical indicators in the rhizospheric soil. Data in (**a**) are presented as means ± SD (*n* = 9). S1P0: PGPR only; S1P1: PASP + PGPR. In (**b**), red and gray gradients denote positive (r > 0) and negative (r < 0) correlations, respectively. Asterisks indicate significance (* *p* < 0.05, *** *p* < 0.001; Pearson’s correlation). S13: bacterial content, NH_4_: Ammonium nitrogen, NO_3_: Nitrate nitrogen, AK: Available potassium, Ure: Soil urease activity.

To investigate the colonization of PGPR strains in the rhizospheric soil of potatoes, we employed an antibiotic-marking method for PGPR and detected the rhizospheric soil from 1 to 21 days after PGPR inoculation. Note that CFU counts (used to quantify bacterial content) were not log-transformed prior to analysis, as the data distribution met the requirements of subsequent statistical tests and untransformed values more intuitively reflect the actual abundance of PGPR in the rhizosphere. The results of bacterial content in the rhizospheric soil showed that in the treatment with PGPR inoculation alone (S1P0), the content in the potato rhizospheric soil gradually decreased over time and eventually stabilized, indicating successful colonization of the strain in the potato rhizospheric soil (Figure 7a). Under the condition of PASP application followed by PGPR inoculation (S1P1), compared to the S1P0 treatment, the initial bacterial content (Day 1) in the potato rhizospheric soil was temporarily reduced; thereafter, as the temporary inhibitory effect diminished, the PGPR content showed an increasing trend, followed by a gradual decline and eventual stabilization, indicating that the strain can colonize the potato rhizospheric soil under PASP application (Figure 7a). To further clarify the causal relationship between the PGPR strain and plant growth as well as nutritional effects, we performed a correlation analysis between PGPR content in the rhizospheric soil and soil biochemical indicators. The results showed that the colonization of the PGPR strain had no significant correlation with NH_4_^+^-N or NO_3_^−^-N but was significantly negatively correlated with AK and S-Ure activity (*p* < 0.05, *p* < 0.001). This further indicates that changes in soil biochemical characteristics are the result of the combined action of the rhizospheric microbial community mediated by PGPR inoculation (Figure 7b).

## 3. Discussion

### 3.1. PGPR Inoculation Enhances Potato Growth Through Rhizosphere Nutrient Modulation

**Figure A2:**
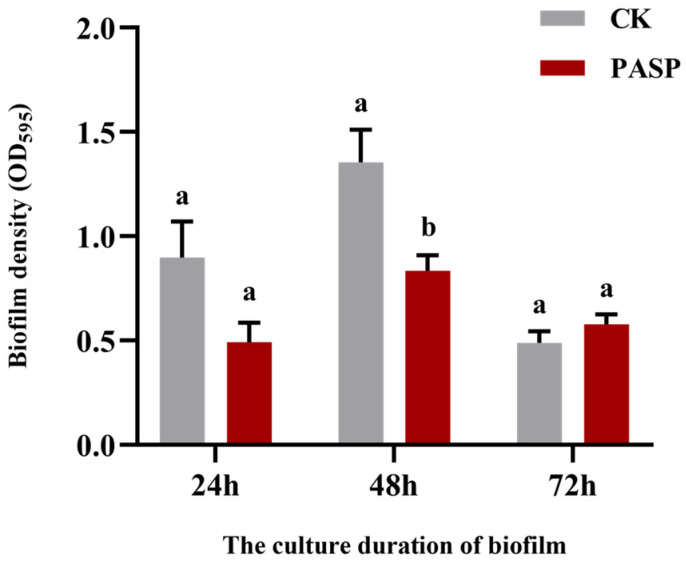
Effect of PASP on biofilm formation of S13 strain. Data are presented as means ± SD (*n* = 6). Different letters indicate significant differences (ANOVA, Duncan’s test, *p* < 0.05).

Most PGPR strains exhibit growth-promoting properties. For instance, *Bacillus* sp. can stimulate plant growth by producing plant hormones such as IAA and solubilizing inorganic phosphorus [21], while *Paenibacillus polymyxa* HL14-3 induces ABA production in cucumbers, enhancing their growth under drought stress [22]. In this study, compared to the control and PASP application alone (S0P1), additional PGPR inoculation (S1P1) further increased plant height, stem diameter, root length, and dry matter accumulation of potatoes (Figure 1), confirming that introducing PGPR into a PASP-amended environment provides an additional growth-promoting effect. However, the growth promotion in this combined treatment was lower than that in the PGPR-only treatment (S1P0), indicating that the interaction between PASP and PGPR is not simply synergistic. Monitoring of PGPR colonization dynamics provided a key mechanistic clue: the initial colonization (Day 1) of PGPR was transiently inhibited in the combined treatment (Figure 7a), which aligned with the transient negative impact of PASP on the biofilm formation of the inoculated strain (Figure A2). This initial suppression might have partially compromised the immediate growth-promoting capacity of the PGPR, thereby explaining part of the performance difference between the combined and PGPR-only treatments. Nevertheless, as the inhibitory effect of PASP diminished, the PGPR content subsequently recovered and eventually stabilized (Figure 7a), indicating that the strain could adapt to the PASP environment and successfully colonize, laying a biological foundation for its sustained growth-promoting function in the PASP-modified rhizosphere micro-ecology.

To further investigate the mechanism from a nutrient perspective, we measured the nutrient content in the rhizosphere soil and analyzed its correlation with dry matter accumulation in different plant parts. The results indicated that the S1P1 treatment significantly increased the levels of NH_4_^+^-N, NO_3_^−^-N, and AK compared to the S0P1 treatment (Figure 2), with a significant positive correlation observed between AK content and root dry matter accumulation (Figure 5). Mechanistically, plant growth and development are strongly correlated with rhizosphere nutrient availability. For example, soil nitrogen content influences the absorption and utilization of potassium in rapeseed (*Brassica napus* L.) [23], suggesting potential crosstalk between nitrogen and potassium assimilation pathways that may underpin the observed growth promotion. Soil potassium content can alter root architecture, under low potassium conditions, tea plant (*Camellia sinensis* L. Kuntze) roots secrete organic acids to solubilize potassium salts, whereas under high potassium conditions, they upregulate genes related to cellulose degradation and potassium transporters, thereby increasing total root length and fine root proportion in response to soil potassium gradients [24]. These findings suggest that the increased biomass of potatoes following PGPR inoculation under PASP application may be linked to the further enhancement of soil available nitrogen and potassium content, which may have triggered root morphological and physiological responses, thereby enhancing nutrient acquisition.

### 3.2. The Changes in Soil Biochemical Properties Were Likely Mediated by PGPR Inoculation via Alterations in the Rhizosphere Microbial Community

PGPR can enhance nutrient availability in the plant rhizosphere, increasing the content of available nitrogen, potassium, and phosphorus [25]. For example, *Azoarcus* sp. BH72 has been shown to increase nitrogen content in the rhizosphere of kallar grass (*Leptochloa fusca* L. Kunth) under low nitrogen conditions [26], while *Pseudomonas* sp. and *Bacillus* sp. can secrete oxalic acid, citric acid, and phytase to solubilize organic or inorganic phosphorus, thereby enhancing its availability to plants [27]. In this study, PGPR inoculation under PASP application significantly increased the levels of NH_4_^+^-N, NO_3_^−^-N, AK, and S-Ure activity in the rhizosphere soil of potatoes (Figure 2), which is consistent with previous findings. Furthermore, Figure 6 and Figure 7 revealed that the changes in soil biochemical characteristics within the potato rhizosphere were closely associated with the rhizosphere microbial community mediated by PGPR inoculation. RDA combined with VPA indicated that AK content was the most significantly affected parameter, followed by NH_4_^+^-N and NO_3_^−^-N content (Figure 6a,b). The lack of a significant positive correlation, or even the presence of negative correlations, between the colonization density of the PGPR strain and these key soil nutrient indices (Figure 7b) further suggests that the observed changes in soil biochemistry were not directly driven by the inoculated strain alone, but rather by the collective action of the restructured microbial community, with PGPR inoculation potentially serving as an initiating trigger.

**Figure A3:**
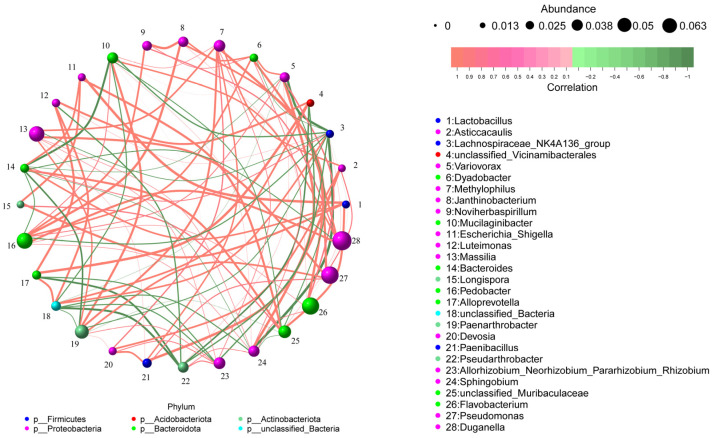
Co-occurrence network analysis of rhizosphere microbial communities between S0P1 and S1P1 treatments (Correlation type: Pearson; Correlation coefficient threshold: 0.5; Correlation *p*-value threshold: 0.05). Nodes represent microbial taxa, with the node size proportional to the relative abundance and the node color indicating the phylum-level taxonomy. Edges represent significant correlations between taxa, with the edge thickness proportional to the correlation strength and the edge color denoting positive (red) or negative (green) correlations.

Subsequent correlation analysis between microbial taxa and edaphic factors showed that these changes were associated with specific functional taxa, such as *Paucibacter* (a genus recognized for its metabolic versatility in carbon cycling and xenobiotic degradation), *Massilia* (a rhizosphere-competent genus involved in root exudate utilization and phytohormone modulation), and *Methylophilus* (a methylotrophic bacterium contributing to single-carbon metabolism and nitrogen mineralization) (Figure 6c). Moreover, microbial co-occurrence network analysis further revealed predominant positive correlations among these key functional taxa (Figure A3), suggesting synergistic interactions within the consortium. Studies have shown that the addition of exogenous PGPR can increase the abundance of beneficial microorganisms in the microbial community, thereby promoting plant growth [28]. For example, the use of *Bacillus velezensis* SQR9 increased the relative abundance of *Pseudomonas* sp. and *Bacillus* sp. in the rhizosphere of cucumber (*Cucumis sativus* L.) and promoted cucumber growth in association with the native strain *Pseudomonas stutzeri* [29]. Inoculation with *Bacillus velezensis* containing agricultural waste enhanced the biofilm formation and colonization ability of native *Pseudomonas fluorescens*, promoting strawberry (*Fragaria × ananassa*) growth [30]. In line with this, the shifts in microbial community composition observed in our study may result from both the direct colonization of *E. asburiae* S13 and its indirect effects on the native microbiota via alterations in root exudates. This suggests that changes in rhizosphere soil nutrient content are not solely attributed to a single microbial species but are likely the result of the combined action of rhizosphere microbial communities mediated by exogenous PGPR.

### 3.3. The Introduction of Exogenous Microorganisms Has the Potential to Reshape the Structure of the Rhizosphere Soil Microbial Community

Previous studies have demonstrated that the use of microbial inoculants can not only directly affect host plants but also regulate the structure and composition of soil microbial communities, thereby synergistically promoting plant growth, disease resistance, and stress tolerance [28]. In this study, PGPR inoculation under PASP application reduced microbial richness and altered β-diversity (Figure 3), indicating that PGPR inoculation significantly impacted the soil microbial community structure, consistent with previous findings. This restructuring may be driven by resource competition, as the inoculated *E. asburiae* S13 may occupy overlapping ecological niches with native taxa, potentially explaining the decrease in *Proteobacteria*, *Firmicutes*, and *Actinobacteria*. The introduction of exogenous microorganisms may stimulate the host plant to produce specific secretions that favor their colonization, enhancing their competitive advantage in nutrient and ecological niche competition [31,32]. For instance, inoculation with *Pseudomonas simiae* WCS417 can induce the secretion of scopoletin by Arabidopsis (*Arabidopsis thaliana* L. Heynh) roots, facilitating the colonization of this strain while inhibiting the growth of pathogens and other native microbial communities [33]. Additionally, PGPR inoculation increased the abundance of phyla such as *Bacteroidota* and *Acidobacteriota* in the potato rhizosphere soil, as well as the abundance of genera like *Paucibacter* and *Massilia*. Random forest analysis revealed that the increased abundance of genera such as *Turicibacter* and *Rhodanobacter* significantly influenced the differences in microbial community composition between groups (Figure 4). These increased microbial populations often exhibit strong growth-promoting and soil nutrient-activating abilities. For example, *Massilia* strains JJY03 and JJY04 can solubilize phosphate and produce IAA [34], while *Rhodanobacter* strains Si-c and S2-g demonstrate robust phosphate solubilization and nitrogen fixation capabilities [35]. This suggests that PGPR inoculation may promote the recruitment of beneficial microorganisms associated with growth promotion, contributing to the restructuring of the potato rhizosphere microbial community.

Notably, the presence of PASP, with its unique biodegradable and aspartic acid-releasing properties [3,4,5,6], likely created a distinct rhizosphere chemical environment compared to other common synergists like humic acids, thereby specifically modulating the colonization activity of the inoculant and the interactions within the microbial community under this condition (Figure 7 and Figure A2). Furthermore, the degradation products of PASP, such as aspartic acid, could act as microbial signaling molecules or nutrients [4,6], providing a feedback mechanism that further influences community assembly and function.

It is important to note, however, that the introduction of exogenous *Enterobacter* species carries potential ecological risks, such as unintended impacts on native soil ecosystems, which warrant consideration in future applications. Moreover, the outdoor environmental conditions during the experiment may have also contributed to the observed microbial community dynamics. While the effects observed here are specific to the *E. asburiae* S13 strain, the general principle of PASP facilitating PGPR-mediated microbiome restructuring may be applicable to other PGPR strains, though the specific outcomes would depend on the functional traits and ecological preferences of the introduced strain, an area requiring further investigation. From a practical perspective, this PASP-PGPR combination strategy demonstrates potential to reduce reliance on chemical fertilizers, offering a promising approach for sustainable potato production.

## 4. Materials and Methods

### 4.1. Experimental Site and Materials

The pot experiment was conducted at the experimental base of Sichuan Agricultural University, Chengdu, Sichuan, China (30°43′ N, 103°52′ E; altitude 538 m), which is situated in a mid-subtropical humid monsoon climate zone with a multi-year average temperature of 16.7 °C, precipitation of 771.2 mm, sunshine duration of 1130.7 h, and a frost-free period of over 281 d. The experiment ran from late December 2023 to May 2024, during which the plants were exposed to natural variations in light and temperature. The commercial potato variety used was “Chuanyu 50”, a medium-late maturing variety with vigorous field growth. PASP (molecular weight 10 kDa, purity: Analytical Reagent [AR], active component > 99.7%, nutrient-free) was purchased from Zhengzhou Guanda Chemical Products Co., Ltd. (Zhengzhou, China). The fertilizer applied was compound fertilizer (N: P_2_O_5_: K_2_SO_4_ = 16: 6: 8; total nutrients ≥ 40%; Shanxi Tianji Coal Chemical Group Co., Ltd., Taiyuan, China). The PGPR strain S13, identified as *E. asburiae* through sequencing of the 16S rRNA gene V3-V4 region, with its identity confirmed by phylogenetic analysis showing 96% similarity to *E. asburiae* strains W6-2 and W6-3 [36], and deposited in the NCBI database under accession number MH883312, was isolated and preserved by the Key Laboratory of Crop Cultivation and Farming System, College of Agriculture, Sichuan Agricultural University. Previous studies confirmed that strain S13 can produce IAA and siderophores, and solubilize phosphorus and potassium [36].

To subsequently determine the colonization of this strain in the potato rhizosphere, antibiotic resistance marking of strain S13 was performed based on its annotated drug resistance genes from genomic information [37]. This procedure was approved by the Institutional Biosafety Committee of Sichuan Agricultural University. Azithromycin (a macrolide antibiotic), Rifampicin (a rifamycin antibiotic), and Tetracycline were selected from the drugs for which the strain had annotated tolerance genes [36]. Specifically, the activated bacterial suspension was serially diluted and spread on LB antibiotic plates containing the respective antibiotics (Azithromycin 50 µg mL^−1^; Rifampicin 25 µg mL^−1^; Tetracycline 5 µg mL^−1^). Single colonies growing on these plates were picked and inoculated onto selective LB plates containing all three antibiotics. Single colonies were then streak-purified for five consecutive generations on LB agar plates before being stored in glycerol at −20 °C for later use.

The preparation of the *E. asburiae* S13 bacterial suspension and the selection of the working concentration followed the method described by Shi [36]. Briefly, a single activated colony was inoculated into LB broth and cultured at 37 °C with shaking at 170 rpm for 24 h. The resulting suspension was adjusted to 1 × 10^8^ CFU mL^−1^ (OD_600_ = 0.8–1.0) using sterile water, and then diluted 1:10,000 before application.

### 4.2. Pot Experiment Design

The experiment followed a completely randomized design with four treatments: (1) untreated control (CK); (2) PGPR inoculation only (S1P0); (3) PASP application without PGPR inoculation (S0P1); (4) combined application of PASP and PGPR inoculation (S1P1). Each treatment consisted of 25 pots, with one seed potato sown per pot at a depth of approximately 10 cm. The soil used for the pots was collected from the experimental field, where the previous crop was rice. The baseline soil nutrient content was organic matter 25.20 g kg^−1^, ammonium nitrogen 11.34 mg kg^−1^, nitrate nitrogen 19.35 mg kg^−1^, available phosphorus 27.01 mg kg^−1^, and available potassium 86.99 mg kg^−1^, and its texture was classified as silty loam. All pots received 10 g of compound fertilizer based on the unit nutrient requirements of field-grown potato plants. PASP (0.5 g per pot, equivalent to 5% of the fertilizer mass), applied at this dosage based on preliminary optimization experiments that demonstrated its efficacy in enhancing nutrient availability and promoting potato plant growth, was dissolved in sterile water and applied once at sowing along with the fertilizer. Inoculation with the *E. asburiae* S13 suspension was performed via root drenching. Specifically, when approximately 70% of the plants per treatment had sprouted, 30 mL of the bacterial suspension was applied per plant to the S1P0 and S1P1 groups, while an equal volume of sterile water was applied to the CK and S0P1 groups. Fertilization and watering were conducted after sowing, and regular irrigation and manual weeding were performed throughout the growth period.

### 4.3. Sample Collection and Processing

#### 4.3.1. Plant Samples

At the potato seedling stage (approximately 25 days after sprouting), samples were collected. Six healthy, pest-free, and uniformly growing plants were selected from each treatment group for phenotypic image capture and growth parameter analysis, including plant height, stem diameter, root length, and dry weight measurement.

#### 4.3.2. Rhizosphere Soil Samples

Rhizosphere soil, operationally defined as soil tightly adhering to the root surface after gentle shaking, typically within 1–3 mm of the root interface, was collected at 1, 3, 7, 14, and 21 days after the application of the *E. asburiae* S13 suspension to determine the colonization density of strain S13. For each sampling, three pots of plants with similar growth were selected. The soil around the stem base was gently loosened, and plants were carefully excavated to avoid root damage. After shaking off loosely adhering soil, soil tightly adhering to the roots was collected using a sterile brush. The soil collected from the three plants was mixed and then divided into three subsamples for immediate determination of colonization density.

Soil samples for rhizosphere microbial community analysis and soil biochemical analysis were collected from the six plants used for growth indicator measurements. After shaking off loosely adhering soil, the soil tightly adhering to the roots was brushed off and mixed. This composite sample was divided into two parts: one part was flash-frozen in liquid nitrogen and stored at −80 °C for microbial analysis, and the other part was air-dried, ground, and passed through a 60-mesh sieve for biochemical analysis.

### 4.4. Experimental Methods

#### 4.4.1. Plant Growth Indicators and Dry Matter Determination

Plant height and root length were measured using a meter ruler. Plant height was recorded from the stem base to the apical bud, and root length was measured from the root base to the tip of the longest root. Stem diameter was measured at the base using a vernier caliper, taking the average of the widest and narrowest diameters. For dry matter determination, roots, stems, and leaves were separated, dried in an oven at 105 °C for 30 min, and then dried at 80 °C until constant weight was achieved before weighing.

#### 4.4.2. Soil Biochemical Analysis

NH_4_^+^-N: Extracted using 2 M KCl and quantified via indophenol blue colorimetry (UV-756 spectrophotometer, Shanghai Precision Scientific Instrument Co., Ltd., Shanghai, China; 625 nm) [38].

NO_3_^−^-N: Extracted with 2 M KCl, acidified with 10% H_2_SO_4_, and quantified using dual-wavelength spectrophotometry (UV-756 spectrophotometer, Shanghai Precision Scientific Instrument Co., Ltd., Shanghai, China; 220 nm and 275 nm) [39].

AK: Extracted with 1 M CH_3_COONH_4_ (pH 7.0) and analyzed using flame photometry (FP6400, Shanghai Jingke Industrial Co., Ltd., Shanghai, China) [40].

S-Ure activity: Measured using a commercial kit (Cat#ADS-F-TR001, Jiangsu Adison Biotechnology Co., Ltd., Taizhou, China) according to the manufacturer’s instructions.

#### 4.4.3. PGPR Strain Colonization Quantification

Referring to the method of Xiang [37], 1 g of each soil sample was added to 10 mL of sterile water, vortexed for 2 min, and allowed to stand for 30 min. The soil suspension was serially diluted with sterile water, and 100 µL aliquots from each dilution were spread onto selective LB plates containing all three antibiotics, with three replicates per dilution. The plates were sealed and incubated at 37 °C for 48 h, after which the developed colonies were counted.

#### 4.4.4. Rhizosphere Microbiome Sequencing of Potato

Soil microbial communities from the key treatments S0P1 and S1P1 were analyzed, with nine biological replicates per treatment. Total genomic DNA was extracted from soil samples using the TGuide S96 Magnetic Soil/Stool DNA Kit (Tiangen Biotech Co., Ltd., Beijing, China). The V3-V4 hypervariable region of the bacterial 16S rRNA gene was amplified with primers 338F (5′-ACTCCTACGGGAGGCAGCA-3′) and 806R (5′-GGACTACHVGGGTWTCTAAT-3′). PCR products were analyzed by agarose gel electrophoresis and purified using the D6492-00 OMEGA Cycle Pure Kit (Noblebio Technology Co., Ltd., Beijing, China). Paired-end sequencing (2 × 250 bp) was performed on the Illumina NovaSeq 6000 platform (Illumina, San Diego, CA, USA), yielding an average depth of 50,000 reads per sample. Raw sequences were processed as follows: first, quality filtering and adapter removal were performed using Trimmomatic (v0.33) (quality cutoff: Q20) and Cutadapt (v1.9.1), respectively; then, the dataset was rarefied to 60,000 reads per sample; finally, denoising, sequence assembly, and chimera removal were conducted using the DADA2 plugin in QIIME2 (v2023.2), generating high-quality amplicon sequence variants (ASVs) for subsequent analysis.

### 4.5. Statistical Analysis

Plant growth parameters and soil biochemical properties were analyzed using one-way ANOVA followed by Duncan’s multiple range test (*p* < 0.05) in SPSS Statistics 26. Data visualization was performed using GraphPad Prism 9.5.

**Figure A4:**
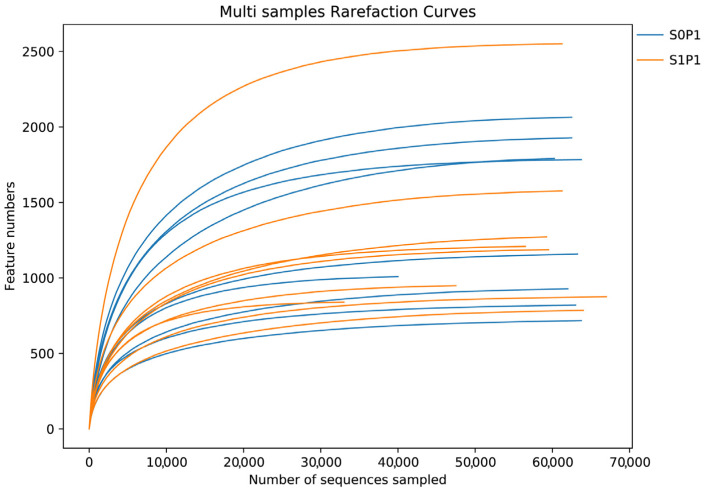
Multi samples Rarefaction Curves for S0P1 and S1P1 treatments.

For microbial community analysis, α-diversity indices (Chao1 and Shannon) were calculated using QIIME2 (v2023.2). Rarefaction curves were analyzed to confirm sufficient sequencing depth (Figure A4). OTUs were clustered at a 97% similarity threshold using USEARCH (v10.0) implemented in R, with low-abundance OTUs (<0.005% of total sequences) filtered out. β-Diversity was visualized via PCoA based on Bray–Curtis dissimilarity. Functional profiling of microbial communities was inferred using PICRUSt2 (v2.3.0) against the KEGG database. Differentially abundant KEGG pathways between groups were identified using DESeq2 (v1.40.0) (*p*-value threshold < 0.05). Taxonomic annotation was performed in QIIME2 (v2023.2) with reference to the NCBI database (https://www.ncbi.nlm.nih.gov). Differential abundance of taxa between groups was assessed using Metastats (v0.3.2) (*p* < 0.05). Feature taxa contributing to community differences were identified via random forest analysis using the RandomForest package (v4.6-10) in R (ntree = 500). Relationships between microbial diversity and environmental factors were evaluated through RDA and VPA implemented in the vegan package (v2.3) in R.

## 5. Conclusions

In this study, the inoculation of PGPR under PASP application led to a significant restructuring of the rhizosphere microbial community composition and architecture in potato, along with the selective enrichment of beneficial microbial taxa. The PGPR-mediated microbial consortia collectively enhanced soil nutrient availability, as evidenced by notable increases in NH_4_^+^-N, NO_3_^−^-N, and AK concentrations, along with elevated S-Ure activity in the rhizosphere microenvironment. These synergistic improvements in soil biochemical properties resulted in measurable physiological outcomes, such as increased stem and root biomass, thus demonstrating substantial growth promotion effects. Overall, our preliminary findings indicate that PGPR inoculation under PASP supplementation represents an effective agroecological strategy to enhance plant growth promotion. However, the experiment was limited to pot conditions, and the lack of field validation may restrict the practical extrapolation of conclusions. Although correlation analysis has revealed associations between microbial communities and nutrients, future studies involving field validation and metagenomic functional profiling are needed to solidify these findings and provide deeper mechanistic insights.

## Data Availability

The datasets generated and analyzed during the current study are available from the corresponding author upon reasonable request due to privacy.
